# Comparison of Data Mining Methods for the Signal Detection of Adverse Drug Events with a Hierarchical Structure in Postmarketing Surveillance

**DOI:** 10.3390/life10080138

**Published:** 2020-08-05

**Authors:** Goeun Park, Heesun Jung, Seok-Jae Heo, Inkyung Jung

**Affiliations:** Division of Biostatistics, Department of Biomedical Systems Informatics, Yonsei University College of Medicine, 50-1 Yonsei-ro, Seodaemun-gu, Seoul 03722, Korea; gogoeun@yuhs.ac (G.P.); 6549209@naver.com (H.J.); sjheo@yuhs.ac (S.J.H.)

**Keywords:** disproportionate reporting rate, drug safety surveillance, pharmacoepidemiology, spontaneous reporting system, tree-based scan statistic

## Abstract

There are several different proposed data mining methods for the postmarketing surveillance of drug safety. Adverse events are often classified into a hierarchical structure. Our objective was to compare the performance of several of these different data mining methods for adverse drug events data with a hierarchical structure. We generated datasets based on the World Health Organization’s Adverse Reaction Terminology (WHO-ART) hierarchical structure. We evaluated different data mining methods for signal detection, including several frequentist methods such as reporting odds ratio (ROR), proportional reporting ratio (PRR), information component (IC), the likelihood ratio test-based method (LRT), and Bayesian methods such as gamma Poisson shrinker (GPS), Bayesian confidence propagating neural network (BCPNN), the new IC method, and the simplified Bayesian method (sB), as well as the tree-based scan statistic through an extensive simulation study. We also applied the methods to real data on two diabetes drugs, voglibose and acarbose, from the Korea Adverse event reporting system. Only the tree-based scan statistic method maintained the type I error rate at the desired level. Likelihood ratio test-based methods and Bayesian methods tended to be more conservative than other methods in the simulation study and detected fewer signals in the real data example. No method was superior to the others in terms of the statistical power and sensitivity of detecting true signals. It is recommended that those conducting drug‒adverse event surveillance use not just one method, but make a decision based on several methods.

## 1. Introduction

It is critical to detect signals of adverse drug reactions from real-world data early enough to protect public health. From the real-world data, we could identify new effects of drugs that had not been identified during premarketing clinical trials. Adverse event (AE) information after drug marketing is often collected via a spontaneous reporting system to identify any long-term adverse drug reactions. In Korea, for example, the Korea Institute of Drug Safety and Risk Management (www.drugsafe.or.kr) collects the information through a spontaneous reporting system.

Through this system, anyone, for example, a patient who has taken the drug, a doctor, or the manufacturer, can report an AE. They report information such as the symptoms of the AE, the date of onset, the name of the drug, the frequency and duration of the dose, patient information, and causality assessment information. As the causality can only be reported by medical experts, the information reported by the patients does not confirm that an AE has been caused by a particular drug. In addition, there could be issues related to data quality and under-reporting. The total number of people who received the drug and the number of AEs are not precisely known. It is difficult to determine a causal relationship between drugs and adverse effects from a spontaneous reporting system database. We can only identify signals of adverse drug reactions, so additional in-depth studies are needed [1].

Statistical analysis is performed to detect whether any particular AEs have occurred more frequently or whether there are any unexpected AEs. Among the various data mining tools, disproportionality methods are widely used for AE signal detection. Different disproportionality methods exist based on different measures such as the reporting odds ratio (ROR) [2], proportional reporting ratio (PRR) [3], information component (IC), likelihood ratio test-based method (LRT) [4], gamma Poisson shrinker (GPS) [5], Bayesian confidence propagating neural network (BCPNN) [6], new IC method [7,8], and the simplified Bayesian method (sB) [9]. ROR, PRR, IC, and LRT are frequentist methods, while GPS, BCPNN, the new IC method, and sB are Bayesian methods [10]. Some studies suggest that Bayesian methods, such as multi-item gamma Poisson shrinker (MGPS) and BCPNN, outperform frequentist methods such as PRR [2,3,6,7,11]. Other studies showed that the sB method performed better than BCPNN and PRR [9,11]. Unlike other methods, the GPS method needs to estimate hyperparameters of the prior distribution using the whole data. Because of this process, the GPS method requires more computation time. Thus, most pharmaceutical companies and national and international pharmacovigilance organizations use other methods more often than the GPS method [12].

Another type of data mining method for signal detection is the tree-based scan statistic (TreeScan) proposed by Kulldorff et al. [13]. This method simultaneously searches for signals at any level (or layer) of AE in a hierarchical structure, adjusting for the multiple testing problem. It has been applied to drug safety surveillance as well as occupational disease surveillance [13,14].

Both LRT and TreeScan methods were developed based on a likelihood ratio test with the test statistic as the maximum likelihood ratio. Moreover, both methods use the Monte Carlo method to obtain the empirical distribution for statistical inference. The LRT method can handle AEs such as system-organ classes (SOC), preferred terms (PT), or included terms (IT) (only one layer, not all the layers together). If the AE is coded as PT, the LRT method detects the signal of single PT. The TreeScan method for detecting AE signals for a fixed drug in data with multiple layers may consider all the layers, and search for signals of PT and SOC (or other layers) together. The LRT method is more general and covers different aspects of safety signal detection. The TreeScan method is for detecting AE or certain prespecified AE groups as signals for a fixed drug, which is a special case of the LRT method.

Clinical information related to adverse drug reactions is generally coded using medical dictionaries such as the World Health Organization Adverse Reaction Terminology (WHO-ART) and the Medical Dictionary for Regulatory Activities (MedDRA), which have a hierarchical structure. There are several studies comparing the performance of disproportionality methods in a usual database format with AEs and drug combination data [2,3,6,7,9,11,12,15]. However, it is not clear which method performs better than the others when AEs are classified into a hierarchical structure. There is a study comparing the application of the GPS and TreeScan methods to real cohort data by Brown et al. [15]. They showed that the signaled regions were similar. However, in some cases, the TreeScan method detected signals that were not detected by the GPS. We do not know which results are more reliable.

The purpose of this study was to compare the performance of several different data mining methods for signal detection in adverse drug event data grouped into hierarchical structures. Through an extensive simulation study, we evaluated the performance of ROR, PRR, IC, LRT, GPS, BCPNN, sB, and TreeScan on datasets generated based on the WHO-ART’s hierarchical structure. Originally, the methods except TreeScan were not developed for hierarchically structured data. We evaluated all the methods by considering all layers, instead of limiting to a single layer, to better reflect the hierarchical data structure and make fair comparisons. We used the type I error rate, power, sensitivity, and positive predictive value as performance measures. We also compared the application results of the methods to a real dataset from the Korea adverse event reporting system (KAERS).

## 2. Signal Detection Method

Several different data mining methods have been developed to detect unusually high disproportionate reporting rates from the large drug safety databases. In this paper, we considered ROR, PRR, IC, LRT, GPS, BCPNN, new IC, sB, and TreeScan, which have been relatively widely used. We mainly referred to Huang et al. [10] for a review of the methods apart from TreeScan.

From a large drug safety database, the number of AEs by drugs can be presented in matrix form with I rows of AEs and J columns of drugs. For a particular AE (*i*th AE) at a particular drug (*j*th drug), the data can be summarized in a 2 × 2 table, as shown in Table 1.

### 2.1. Frequentist Methods

#### 2.1.1. Reporting Odds Ratio (ROR)

The ROR is the odds ratio that a particular AE is reported in patients who take a specific drug compared to patients who take other drugs [2]. The ROR for the *i*th AE and the *j*th drug ($ROR_{ij}$) is estimated as

$$R\hat{O}R_{ij}=\frac{n_{ij}/\left(n_{i.}-n_{ij}\right)}{\left(n_{.j}-n_{ij}\right)/\left(n_{..}-n_{i.}-n_{.j}+n_{ij}\right)}=\frac{n_{ij}\left(n_{..}-n_{i.}-n_{.j}+n_{ij}\right)}{\left(n_{i.}-n_{ij}\right)\left(n_{.j}-n_{ij}\right)}$$

and if either $\left(n_{i.}-n_{ij}\right)$ or $\left(n_{.j}-n_{ij}\right)$ is equal to 0, then $R\hat{O}R_{ij}$ is not defined. The log-transformed $R\hat{O}R_{ij}$ can be approximated to a normal distribution as follows:

$$\log\left(R\hat{O}R_{ij}\right)\;\dot{\sim }\;N\left(\log\left(ROR_{ij}\right),\sigma_{ROR_{ij}}^{2}\right),$$

$${\hat{\sigma }}_{ROR_{ij}}^{2}\approx \frac{1}{n_{ij}}+\frac{1}{\left(n_{i.}-n_{ij}\right)}+\frac{1}{\left(n_{.j}-n_{ij}\right)}+\frac{1}{\left(n_{..}-n_{i.}-n_{.j}+n_{ij}\right)}\;.$$

We can obtain an approximate 100(1−α)% confidence interval (CI) for $ROR_{ij}$ as:

$$CI_{ROR_{ij},100\left(1-\alpha \right)\%}=\exp\left(\log\left(R\hat{O}R_{ij}\right)\pm z_{1-\alpha /2}\sqrt{{\hat{\sigma }}_{ROR_{ij}}^{2}}\right),$$

where $z_{1-\alpha /2}=\Phi \left(1-\alpha /2\right)$ and Φ is the standard normal distribution’s cumulative distribution function.

The null and the alternative hypotheses to test whether the *i*th AE for the *j*th drug is a signal or not are expressed as:

$$H_{0}\;:ROR_{ij}=1\;\mathrm{vs}.\;H_{a}\;:ROR_{ij}>1.$$

As Evans et al. [3] suggested, the lower bound of $CI_{ROR_{ij},100\left(1-\alpha \right)\%}>2$, so we reject the null hypothesis and conclude that the *i*th AE can be interpreted as a signal of the disproportionate rate (SDR) for the *j*th drug.

#### 2.1.2. Proportional Reporting Ratio (PRR)

The PRR is the ratio of the proportion of patients who reported a particular AE after taking a specific drug to the proportion of patients who have taken other drugs that reported the same AE [3]. We estimate the PRR for the *i*th AE and the *j*th drug ($PRR_{ij}$) as:

$$P\hat{R}R_{ij}=\frac{n_{ij}/n_{i.}}{\left(n_{.j}-n_{ij}\right)/\left(n_{..}-n_{i.}\right)}=\frac{n_{ij}\left(n_{..}-n_{i.}\right)}{n_{i.}\left(n_{.j}-n_{ij}\right)}.$$

If $\left(n_{i.}-n_{ij}\right)\approx n_{i.}$ and $\left(n_{..}-n_{i.}-n_{.j}+n_{ij}\right)\approx \left(n_{..}-n_{i.}\right)$, then $R\hat{O}R_{ij}\approx P\hat{R}R_{ij}$. We use the normal approximation for the distribution of $\log\left(P\hat{R}R_{ij}\right)$ for inference as follows:

$$\log\left(P\hat{R}R_{ij}\right)\;\sim \;N\left(\log\left(PRR_{ij}\right),\sigma_{PRR_{ij}}^{2}\right),$$

$${\hat{\sigma }}_{PRR_{ij}}^{2}\approx \frac{1}{n_{ij}}-\frac{1}{n_{i.}}+\frac{1}{\left(n_{.j}-n_{ij}\right)}-\frac{1}{\left(n_{..}-n_{i.}\right)}.$$

Therefore, an approximate 100(1−α)% CI for $PRR_{ij}$ is expressed as follows:

$$CI_{PRR_{ij},100\left(1-\alpha \right)\%}=\exp\left(\log\left(P\hat{R}R_{ij}\right)\pm z_{1-\alpha /2}\sqrt{{\hat{\sigma }}_{PRR_{ij}}^{2}}\right).$$

The null hypothesis of $H_{0}\;:PRR_{ij}=1$ is rejected if the lower bound of $CI_{PRR_{ij},100\left(1-\alpha \right)\%}>2$, as for PRR.

#### 2.1.3. Information Component (IC)

The IC is based on the relative reporting rate $RR_{ij}$, which indicates how many particular events were reported in excess for a specific drug over the expected number of reported counts under the null hypothesis that a drug and AE are independent. The relative reporting rate is estimated by $\frac{n_{ij}}{E_{ij}}$, where $E_{ij}=\frac{n_{i\cdot }n_{\cdot j}}{n_{\cdot \cdot }\;}$ is the expected number of reports for the *i*th AE and the *j*th drug under the null hypothesis. The IC for the *i*th AE and the *j*th drug is defined as follows:

$$IC_{ij}=\log_{2}RR_{ij}=\frac{\log\left(RR_{ij}\right)}{\log2}.$$

The $IC_{ij}$ is estimated as ${\hat{IC}}_{ij}=$ $\log_{2}\frac{n_{ij}}{E_{ij}}$ for $n_{ij}>0,\;n_{i\cdot }>0,$ and $n_{\cdot j}>0$. The estimated variance of ${\hat{IC}}_{ij}$ is given by ${\hat{\sigma }}_{{\hat{\mathrm{IC}}}_{ij}}^{2}≒\frac{1}{{\left(\log2\right)}^{2}\;}\left\{\frac{1}{n_{ij}}+\frac{1}{n_{i\cdot }}+\frac{1}{n_{\cdot j}\;}\right\}$. An approximate 100(1−α)% CI for $IC_{ij}$ is expressed as follows:

$$CI_{100\left(1-\alpha \right)\%}=\exp\left(\log\left({\hat{IC}}_{ij}\right)\pm z_{1-\frac{\alpha }{2}}\sqrt{{\hat{\sigma }}_{{\hat{\mathrm{IC}}}_{ij}}^{2}}\right).$$

If the lower bound of $CI_{100\left(1-\alpha \right)\%}$> 1, the *i*th AE can be interpreted as a signal.

#### 2.1.4. Likelihood Ratio Test-Based Method (LRT)

Huang et al. [4] proposed the likelihood ratio test statistic, which controls the type I error and false discovery rates by using Monte Carlo hypothesis testing. The null and alternative hypotheses to test whether the *i*th AE for a specific drug ($j^{*})$ is a signal or not are expressed as follows:

$$H_{0}\;:p_{i}=q_{i}\;\mathrm{vs}.\;H_{a}\;:p_{i}>q_{i},$$

where $p_{i}$ and $q_{i}$ are defined as the reporting rates of *i*th AE and other AEs for a specific drug, respectively. The maximum likelihood ratio (MLR) is expressed as follows:

$$MLR=\underset{i}{\max}\left(\frac{{\left(\frac{n_{ij^{*}}}{n_{i.}}\right)}^{n_{ij}}{\left(\frac{n_{.j^{*}}-n_{ij^{*}}}{n_{..}-n_{i.}}\right)}^{n_{j^{*}.}-n_{ij^{*}}}}{{\left(\frac{n_{.j^{*}}}{n_{..}}\right)}^{n_{.j^{*}}-n_{ij^{*}}}}\right)\times I\left({\hat{p}}_{i}>{\hat{q}}_{i}\right),$$

where *I*() is the indicator function, ${\hat{p}}_{i}=n_{ij^{*}}/n_{i.}$, and ${\hat{q}}_{i}=\left(n_{.j^{*}}-n_{ij^{*}}\right)/\left(n_{..}-n_{i.}\right)$. As the distribution of MLR under the null hypothesis is unknown, the Monte Carlo hypothesis testing is used to calculate *p*-values. For details, see Section 2.3.

### 2.2. Bayesian Method

#### 2.2.1. Gamma Poisson Shrinker (GPS)

DuMouchel [5] suggested the GPS method, which is an empirical Bayes signal detection method. The GPS method uses the relative report rate, defined as follows:

$$\lambda_{ij}=\frac{n_{ij}}{E_{ij}},\;\mathrm{where}\;E_{ij}=\frac{n_{i.}\times n_{.j}}{n_{..}}.$$

This indicates the actual frequency compared to the expected frequency. $E_{ij}$ is calculated under the null hypothesis that there is no association between the drug‒AE pairs. The null and alternative hypotheses are expressed as follows:

$$H_{0}\;:\lambda_{ij}=1\;\mathrm{vs}.\;H_{a}\;:\lambda_{ij}>1.$$

The GPS method assumes that the model and prior distributions are as follows:

$$\mathrm{model}\;:n_{ij}|\lambda_{ij}\;\sim^{iid}\;Poisson\left(\mu_{\mathrm{ij}}\right),$$

$$\mathrm{prior}\;:\lambda_{ij}\;\sim \;w\times Gamma\left(\alpha_{1},\beta_{1}\right)+\left(1-w\right)\times Gamma\left(\alpha_{2},\beta_{2}\right),$$

where the observed report count $n_{ij}$ follows the Poisson distribution with unknown mean $\mu_{ij}=E_{ij}\times \lambda_{ij}$. The relative report rate follows the mixture gamma distribution where $Gamma\left(\alpha ,\beta \right)$ is a gamma distribution with mean α/β and variance $\alpha /\beta^{2}$ and 0<w<1 is the prior probability that $\lambda_{ij}$ came from the first gamma distribution of mixture. The hyperparameters $\left(\alpha_{1},\;\beta_{1},\;\alpha_{2},\;\beta_{2},\;w\right)$ are estimated by the empirical Bayes method, which is also known as the maximum marginal likelihood. 

As gamma distribution is a conjugate prior for Poisson distribution, the posterior distribution of $\lambda_{ij}$ can be obtained in a closed form as follows:

$$\mathrm{posterior}\;:\lambda_{ij}|n_{ij}\;\sim \;w_{ij}^{*}\times Gamma\left(\alpha_{1}+n_{ij},\beta_{1}+E_{ij}\right)+$$

$$\left(1-w_{ij}^{*}\right)\times Gamma\left(\alpha_{2}+n_{ij},\beta_{2}+E_{ij}\right),$$

where $w_{ij}^{*}$ is the posterior probability that $\lambda_{ij}$ came from the first gamma distribution of the mixture. This is expressed as follows:

$$w_{ij}^{*}=\frac{w\times f\left(n_{ij}|\alpha_{1},\;\beta_{1},\;E_{ij}\right)}{w\times f\left(n_{ij}|\alpha_{1},\;\beta_{1},\;E_{ij}\right)+\left(1-w\right)\times f\left(n_{ij}|\alpha_{2},\;\beta_{2},\;E_{ij}\right)},$$

where $f\left(n_{ij}|\alpha ,\;\beta ,\;E_{ij}\right)$ is the marginal distribution. This marginal distribution follows the negative binomial distribution as follows:

$$n_{ij}|\alpha ,\beta ,E_{ij}\;\sim \;NB\left(\alpha ,\;\frac{E_{ij}}{E_{ij}+\beta }\right),$$

where $NB(x|r,p)=\left(\begin{array}{c} r+x-1\\ x \end{array}\right)p^{x}{\left(1-p\right)}^{r}$.

The 5th percentile of the posterior distribution of $\lambda_{ij}$ (EB05) is used for decision making. EB05 can be obtained by solving the equation as follows:

$$0.05=\int_{0}^{EB05\left(\lambda_{ij}\right)}f\left(\lambda_{ij}|n_{ij}\right)d\lambda_{ij}.$$

This integral can be solved easily using iterative techniques such as Newton’s method. If EB05($\lambda_{ij}$) is greater than 2, this drug‒adverse effect pair is considered a signal of disproportionate rates (SDR).

#### 2.2.2. Bayesian Confidence Propagation Neural Network (BCPNN)

Bate et al. [6] proposed the BCPNN method based on the IC measure. In the BCPNN method, the IC measure was defined as follows:

$$IC_{ij}=\log_{2}\left(\frac{\theta_{ij}}{\theta_{i.}\times \theta_{.j}\;}\right).$$

The observed reporting counts and marginal counts are assumed to follow a binomial distribution with a beta distribution for priors as follows:

$$n_{ij}|\theta_{ij}\;\sim \;Bin\left(n_{..},\;\theta_{ij}\right)\;\mathrm{with}\;\theta_{ij}\;\sim \;Beta\left(\alpha_{ij},\beta_{ij}\right),$$

$$n_{i.}|\theta_{i.}\;\sim \;Bin\left(n_{..},\;\theta_{i.}\right)\;\mathrm{with}\;\theta_{i.}\;\sim \;Beta\left(\alpha_{i.},\beta_{i.}\right),$$

$$n_{.j}|\theta_{.j}\;\sim \;Bin\left(n_{..},\;\theta_{.j}\right)\;\mathrm{with}\;\theta_{.j}\;\sim \;Beta\left(\alpha_{.j},\beta_{.j}\right),$$

where $\alpha_{ij}=\alpha_{i.}=\beta_{i.}=\alpha_{.j}=\beta_{.j}=1$ and $\beta_{ij}=\frac{1}{E\left(n_{i.}|\theta_{i.}\right)E\left(n_{.j}|\theta_{.j}\right)}-1$. 

Using the delta method, the posterior mean and variance of $IC_{ij}$ can be obtained as follows:

$$E\left(IC_{ij}|data\right)=\log_{2}\frac{\left(n_{ij}+1\right){\left(n_{..}+1\right)}^{2}}{\left(n_{..}+\gamma \right)\left(n_{i.}+1\right)\left(n_{.j}+1\right)},$$

$$Var\left(IC_{ij}|data\right)={\left(\frac{1}{log2}\right)}^{2}\left[\frac{n_{..}-n_{ij}+\gamma -1}{\left(n_{ij}+1\right)\left(1+n_{..}+\gamma \right)}+\frac{n_{..}-n_{i.}+1}{\left(n_{i.}+1\right)\left(n_{..}+3\right)}+\frac{n_{..}-n_{.j}+1}{\left(n_{.j}+1\right)\left(n_{..}+3\right)}\right],$$

where $\gamma ={\hat{\beta }}_{ij}+1=\frac{{\left(n_{..}+2\right)}^{2}\;}{\left(n_{i.}+1\right)\left(n_{.j}+1\right)}$. 

The lower limit of the 95% credible interval for $IC_{ij}$ is calculated by:

$$IC_{\alpha /2}=E\left(IC_{ij}|data\right)-z_{1-\alpha /2}\sqrt{Var\left(IC_{ij}|data\right)},$$

and if $sB_{\alpha ,ij}$ is greater than 2, this drug‒AE pair is a possible signal with a higher reporting rate.

#### 2.2.3. New IC Method

The new IC is an improved method for posterior inference in IC analysis, including an accurate estimate for the mode and significantly improved credibility interval estimates. This method also assumes the number of reports $n_{ij}\sim^{iid}Poisson\left(\lambda_{ij}E_{ij}\right),$ where $\lambda_{ij}$ denotes the relative reporting rate. The prior of parameters $\lambda_{ij}$ is given by $\lambda_{ij}\sim^{iid}Gamma\left(0.5,\;0.5\right),$ and the posterior distribution of $\lambda_{ij}$ is given by $\lambda_{ij}|data\sim^{iid}Gamma\left(n_{ij}+0.5,E_{ij}+0.5\right)$. Then, the New $IC_{ij}$ is the posterior mean of $\log_{2}\lambda_{ij}$, which is $E(\log_{2}\lambda_{ij}|data)≒\log_{2}\frac{n_{ij}+0.5}{E_{ij}+0.5\;}$.

The 95% credible interval limits ($\lambda_{0.025},\;\lambda_{0.975})$ are obtained by:

$$\int_{0}^{\lambda_{\alpha }}Gamma\left(y|n_{ij}+0.5,E_{ij}+0.5\right)dy=\alpha$$

for α = 0.025 and α = 0.975. If the lower limit $\lambda_{0.025}>0$, the *i*th AE can be interpreted as a signal.

#### 2.2.4. Simplified Bayesian

For small datasets, the GPS method is usually not recommended because of instability in the estimation of the hyperparameters. Thus, Huang et al. [9] suggested the simplified Bayesian (sB) method, which assumes a weaker assumption on prior distribution than the GPS method. The sB method uses a single gamma distribution as a prior as follows:

$$\mathrm{prior}\;:\lambda_{ij}\;\sim \;Gamma\left(\alpha ,\alpha \right),$$

with mean 1 and variance 1/α. Huang et al. [9] proposed using three values (0.5, 0.01, and 0.0001) for α. They also called the prior distribution with α=0.5 a less noninformative prior. The other prior distributions were called noninformative priors. The posterior distribution is also a single gamma distribution as follows:

$$\mathrm{posterior}\;:\lambda_{ij}|n_{ij}\;\sim \;Gamma\left(\alpha +n_{ij},\alpha +E_{ij}\right).$$

The lower bound of the 95% credible interval for $\lambda_{ij}$ ($sB_{\alpha ,\;ij}$) is used for detecting signals of SDR. $sB_{\alpha ,\;ij}$ is expressed as follows:

$$E\left(\lambda_{ij}|n_{ij}\right)=\frac{\alpha +n_{ij}}{\alpha +E_{ij}}$$

$$Var\left(\lambda_{ij}|n_{ij}\right)=\frac{\alpha +n_{ij}}{{\left(\alpha +E_{ij}\right)}^{2}}$$

$$sB_{\alpha ,\;ij}=E\left(\lambda_{ij}|n_{ij}\right)-1.645\sqrt{Var\left(\lambda_{ij}|n_{ij}\right)}.$$

If $sB_{\alpha ,ij}$ is greater than 2, this drug‒AE pair is a possible signal with a higher reporting rate. With α=0.5, the sB method is identical to the new IC method [10]. Hence, we only included the sB method in the simulation.

### 2.3. Tree-Based Scan Statistic

In a medical dictionary, all AEs are categorized into a hierarchical tree structure. Kulldorff et al. [13,14] proposed the tree-based scan statistic, which simultaneously searches for signals at any level (or layer) of AEs in a hierarchical structure. We call the last cell of the tree a leaf and the rest a node. That is, the higher level of leaves is the node. A higher-level node is defined as the parent node; the lower level node is defined as the child node. $c_{i}$ is the observed number of AEs for each leaf I and $C=\sum_{i}c_{i}=\sum_{i}n_{ij}$ is the total observed number of AEs reported in patients who have taken a specific drug j and $X=\sum_{i}x_{i}=\sum_{i}n_{i.}$ is the total number of AEs reported in patients who have taken any drugs.

When the branches of a tree are cut, the sum of the observed and total number of AEs in the leaves of each cut, G, $c_{G}=\sum_{i\in G}c_{i}$ and $x_{G}=\sum_{i\in G}x_{i}$, respectively, are obtained. G includes both the child nodes and parent nodes as a unit of AE. For each cut G, we can calculate the log likelihood ratio and test statistic:

$$LR\left(G\right)=c_{G}\log\left(\frac{c_{G}}{x_{G}}\right)+\left(C-c_{G}\right)\log\left(\frac{C-c_{G}}{X-x_{G}}\right).$$

$$T=\underset{G}{\max}LR\left(G\right)\times I\left(\frac{c_{G}}{x_{G}}>\left(\frac{C-c_{G}}{X-x_{G}}\right)\right),$$

where *I*() is the indicator function. The cut G that maximizes LR(G) is the most likely cut of related AEs. The null hypothesis implies that the group defined by cut G has the same ratio of observed to expected AEs as the rest of the tree. In inference, Monte Carlo hypothesis testing is used, calculating the most likely cut in each random dataset. Firstly, the likelihood of the most likely cut in a real dataset is calculated. Secondly, 9999 random datasets are generated under the null hypothesis and the test statistic for each random dataset calculated. Then, the *p*-value is calculated as R/(9999 + 1), where R is the rank of the test statistic of real dataset compared with random datasets.

The LRT and TreeScan methods basically use the same test statistic. Because the TreeScan considers the hierarchical structure in nature, the distribution of the test statistic is also obtained by comparing all possible cuts in the hierarchical structure. Even if the two methods detected the same signal, *p*-values could be different.

## 3. Simulation Study

### 3.1. Data Generation and Evaluation Measures

We generated datasets that reflect WHO-ART’s hierarchical structure, which can be expressed as system-organ classes (SOC), preferred terms (PT), and included terms (IT) for AEs [16]. In the simulation study, we included only SOC and PT levels. To reduce the computation time, we only considered 500 drugs and 300 AEs, which were randomly selected from a total of 2161 PT levels. We followed the approach in the study by Huang et al. [4] to generate our simulation data.

First, we generated marginal counts of AEs $n_{1.},\ldots ,n_{I.}$ (I= 300) and drugs $n_{.1},\ldots ,n_{.J}$ (J= 500) as follows:

$$\left(n_{1.},\ldots ,n_{I.}\right)|n_{..}\;\sim \;Multinomial\left(n_{..},\left(\frac{u_{1}}{\sum_{i=1}^{I}u_{i}},\ldots ,\;\frac{u_{I}}{\sum_{i=1}^{I}u_{i}}\right)\right)\;\left(n_{.j},\ldots ,n_{.J}\right)|n_{..}\;\sim \;Multinomial\left(n_{..},\left(\frac{u_{1}}{\sum_{j=1}^{J}u_{j}},\ldots ,\;\frac{u_{J}}{\sum_{j=1}^{J}u_{j}}\right)\right),$$

where $u\;\sim \;Uniform\left(0,1\right)$ with $n_{..}=\sum_{i=1}^{I}n_{i.}$.

Next, we generated the number of cases reported for a specified drug $\left(j^{*}\right),\;n_{1j^{*}},\ldots ,n_{Ij^{*}}$ using

$$\left(n_{1j^{*}},\ldots ,n_{Ij^{*}}\right)|n_{.j^{*}}\;\sim \;Multinomial\left(n_{.j^{*}},p_{rr}\right),$$

where $p_{rr}=\left(rr_{1j^{*}}\times r_{0}\times \frac{n_{1.}}{n_{..}},\ldots ,\;rr_{Ij^{*}}\times r_{0}\times \frac{n_{I.}}{n_{..}}\right)$ is a vector of probabilities with $rr_{1j^{*}},\ldots ,rr_{Ij^{*}}$ as the relative reporting rates. When $r_{0}$ is considered as the baseline risk, $p_{rr}$ has the constraints that $0\leq rr_{ij^{*}}\times r_{0}\times \frac{n_{i.}}{n_{..}}\leq 1,\;i=1,\ldots ,I$, and $\sum_{i=1}^{I}rr_{ij^{*}}\times r_{0}\times \frac{n_{i.}}{n_{..}}=1$. Note that the number of reported cases was generated for a specific drug, and hence the true signals are signals for each drug. This means that the relative reporting rate for the AE with a true signal is higher than those for all the other AEs for one fixed drug. If an AE is a true signal, the relative reporting rate is greater than 1, while the relative reporting rate is equal to 1 when the AE is a false signal [11]. The cells for the true signals were randomly selected first depending on the assumed proportion of true signals. The relative reporting rate for each of the selected cells as true signals was generated from $Uniform\left(1.2,\;10\right)$ and $Uniform\left(1.2,\;4\right)$.

While the TreeScan method detected signals simultaneously for both SOC and PT levels, all the other methods detected signals from SOC and PT levels separately. To evaluate the performances of the methods considering the hierarchical data structure, we merged two separate results from each level for all methods except the TreeScan method.

We generated 1000 datasets for each of nine different settings with three different total sample sizes (300,000, 500,000, 1,000,000) and three different percentages of true signals (3%, 5%, 10%). We used five different cutoffs, which are the criteria for signal detection for each method. Different criteria have been used depending on the organization for different methods [17]. In practice, one may change the criteria based on experience. We used the same criterion of the lower bound of the 95% CI for fair comparison in our simulation.

To compare the performance, we calculated the type I error rate, sensitivity, positive predicted value (PPV), and power for specific drugs. Under the null hypothesis, the type I error is estimated as follows:

$$\mathrm{Type}\;I\;\mathrm{error}=\frac{\#\;\mathrm{of}\;\mathrm{times}\;\mathrm{detecting}\;\mathrm{at}\;\mathrm{least}\;\mathrm{one}\;\mathrm{false}-\mathrm{positive}\;\mathrm{signal}}{\mathrm{total}\;\#\;\mathrm{of}\;\mathrm{simulated}\;\mathrm{datasets}}.$$

The sensitivity, PPV, and power are estimated as:

$$\mathrm{Sensitivity}=\frac{1}{S}\overset{S}{\underset{s=1}{\sum }}\frac{\#\;\mathrm{of}\;\mathrm{true}-\mathrm{positive}\;\mathrm{signals}\;\mathrm{in}\;s\mathrm{th}\;\mathrm{simulated}\;\mathrm{dataset}}{\#\;\mathrm{of}\;\mathrm{true}\;\mathrm{signals}\;\mathrm{in}\;\mathrm{the}\;s\mathrm{th}\;\mathrm{simulated}\;\mathrm{dataset}}$$

$$\mathrm{PPV}=\frac{1}{S}\overset{S}{\underset{s=1}{\sum }}\frac{\#\;\mathrm{of}\;\mathrm{true}-\mathrm{positive}\;\mathrm{signals}\;\mathrm{in}\;s\mathrm{th}\;\mathrm{simulated}\;\mathrm{dataset}}{\#\;\mathrm{of}\;\mathrm{detected}\;\mathrm{signals}\;\mathrm{in}\;\mathrm{the}\;s\mathrm{th}\;\mathrm{simulated}\;\mathrm{dataset}}$$

$$\mathrm{Power}=\frac{\#\;\mathrm{of}\;\mathrm{times}\;\mathrm{detecting}\;\mathrm{at}\;\mathrm{least}\;\mathrm{one}\;\mathrm{signal}}{\mathrm{total}\;\#\;\mathrm{of}\;\mathrm{simulated}\;\mathrm{datasets}},$$

where S is the total number of simulated datasets with at least one signal detected. We used R software 3.5.2 version (Vienna, Austria) for all simulations and data analyses.

### 3.2. Results

#### 3.2.1. Comparison of Type I Error Rate

To compare the type I error rate of each method and cutoff, all relative reporting rates were set to 1 for each total sample size (Table 2). The type I error rates of the ROR, PRR, and IC methods were relatively high for the standard cutoff and for all total sample sizes, which means that spurious detection could frequently occur even when there are no actual signals. The type I error rates of the GPS and sB methods were close to 0 for the standard cutoff and all total sample sizes. The type I error rates of the ROR, PRR, IC, GPS, BCPNN, and sB methods varied depending on how the cutoff was set. On the other hand, the type I error rates of the LRT and TreeScan methods were close to the prespecified significance level in most cases, although the LRT method had slightly higher type I error rates.

#### 3.2.2. Comparison of Sensitivity, PPV, and Power

Table 3 and Table 4 present the results for sensitivity, PPV, and power of each method when the total sample size is equal to 300,000. The other results are presented in Appendix A. For all simulation settings and the standard cutoff for each method, the ROR, PRR, and IC methods had relatively higher sensitivity and power than the other methods. However, the LRT, GPS, BCPNN, sB, and TreeScan methods had relatively higher PPV than the other methods. This means that the ROR, PRR, and IC methods may detect too many signals regardless of whether they are actually true, so these methods could detect many false signals as well as true ones. On the contrary, the LRT, GPS, BCPNN, sB, and TreeScan methods detected much fewer signals, but more true signals than false ones.

When the relative reporting rates were low (Table 4), all the methods had lower performance compared to when the relative reporting rates were high (Table 3). The GPS, BCPNN, and sB methods had a significant decrease in power and sensitivity, especially the GPS method.

As the percentage of true signals increased for all settings of total sample size, the sensitivity decreased but the PPV and power increased for all methods. As the total sample size increased for all settings of the percentage of true signals, the sensitivity, PPV, and power increased for all methods. However, depending on the cutoff of each method, the sensitivity, PPV, and power varied. No single method was superior to the others overall for all settings.

## 4. Example

### 4.1. Korea Adverse Event Reporting System (KAERS)

The KAERS is a spontaneous reporting system that receives and manages adverse drug events reported by patients, manufacturers, or medicine experts, provided by Korea Institute of Drug Safety and Risk Management. It consists of drugs, AEs, basic demographic, and causality assessment information. When reported, a drug and an AE should be reported together in a pair. These can be reported several times depending on the dose and time. If the same drugs and AEs were reported in duplicate, depending on dose or time, only the first report was counted. Therefore, drugs and AEs are paired only one time.

Causality was assessed at six levels: certain, probable, possible, unlikely, unclassified, and unassessable. The assessment criteria are shown in Table 5. We used all drug‒AE pairs except for ones with an unassessable level. Not only the reported information on a possible causal relationship between an AE and a drug, but also previously unknown or incompletely documented relationships can be a signal. The causality assessment was performed by a reporter, such as a medical institution, expert, manufacturer, pharmacy, or public health center.

In KAERS, AEs were organized under the WHO-ART’s hierarchical structure [16]. This consists of four hierarchical levels: system-organ class (SOC), high-level terms (HLT), preferred terms (PT), and included terms (IT). SOC is the highest level. IT represents various expressions about the same AE in the PT level. HLT is a set of PTs related to each other or having some similar symptoms. HLT may or may not exist and therefore are excluded from the analysis. A small subset of the hierarchical structure is listed in Table 6. However, in the KAERS database, more than half of the reports were reported up to the PT level. Thus, we used the PT level as the lowest level of AEs. In the following illustration, we used the SOC and PT levels in the WHO-ART’s hierarchical structure.

### 4.2. Data

We used drug‒adverse effects pair data from KAERS between 2012 and 2016. Between 2012 and 2016, there were approximately 3.1 million drug‒AE pairs with 1615 kinds of PT-level AEs and 1950 kinds of drugs. Restricting the causality assessment information to certain, probable, possible, unlikely, or unclassified levels, approximately 2.5 million drug‒AE pairs with 1484 kinds of PT level AEs and 1716 kinds of drugs were left. These data contained 32 SOC levels, 1484 PT levels, and 3557 IT levels. Analyses were done with these drug‒AE pairs.

### 4.3. Analysis

We selected two diabetes drugs, voglibose and acarbose, to compare specific results. Both are hypoglycemic agents that are used for type 2 diabetes, along with diet and exercise. These two drugs were selected because of their substantial exposure and comparable characteristics. Voglibose has a simple structure relative to acarbose. Moreover, it is known to be more economical and safer because its absolute administration dose is 1000 times lower than that of acarbose. However, some severe AEs tend to be more reported in voglibose [17,18]. Therefore, we found specific AEs in acarbose and voglibose using KAERS data by the signal detection methods previously described.

First, we compared the number of signals detected by each method from all drug‒adverse effect pairs with 1484 kinds of PT level AEs and 1716 kinds of drugs. Second, the specific signals detected by each method were compared for the two diabetes drugs mentioned above. The detection criteria for each method are shown in Table 7 and the TreeScan method was performed with a simple cut.

### 4.4. Results

Table 8 provides the overall signal detection results of all methods. We used the signal detection criteria presented in Table 7. We summarized the number of detected signals separately for PT and SOC levels. The GPS, BCPNN, and sB methods detected relatively fewer signals than the other methods. The ROR and PRR detected the most signals.

The results of applying all methods to two drugs, voglibose and acarbose, are summarized in Table 9. We report only the AEs that were detected by more than two of the signal detection methods. Voglibose had a higher reported count of all AEs than acarbose. The number of AEs detected by at least one method was higher for voglibose (36 AEs) than for acarbose (31 AEs). For both drugs, the common AEs detected were diarrhea, flatulence, and hypoglycemia at the PT level, and metabolic and nutritional disorders at the SOC level. There was only one common AE detected by all methods in acarbose and voglibose: flatulence at the PT level. Both drugs signaled strongly for flatulence, which is an AE commonly observed in patients with type 2 diabetes [19,20]. In addition, the common AEs detected by all methods were dyspepsia and hypoglycemia at the PT level, and metabolic and nutritional disorders at the SOC level in voglibose. 

## 5. Discussion

A number of disproportionality methods for data mining and the TreeScan method were compared for signal detection during drug surveillance for AEs data grouped into hierarchical structures. We included various frequentist methods such as ROR, PRR, IC, LRT, and TreeScan as well as Bayesian methods such as GPS, BCPNN, and sB. The LRT, GPS, BCPNN, sB, and TreeScan methods detected fewer signals than the ROR, PRR, and IC methods. The power and sensitivity of the GPS, sB, LRT, and TreeScan methods tended to be lower than those of others, which implies that these methods are more conservative. The higher power and sensitivity of the ROR, PRR, and IC methods seemed to be due to the higher type I error rates. The three methods had lower PPV. The TreeScan method controls the type I error rate at the desired level, while other methods cannot control this or find appropriate cutoffs for the desired type I error rate. However, no method was superior to the others in relation to all performance measures.

We observed similar patterns in the analysis results of the KAERS data. The GPS and sB methods detected much fewer signals than the others overall. For the two specific drugs, some common AEs were detected by all methods. The ROR, PRR, and IC methods detected additional signals that were not detected by the GPS, sB, LRT, or TreeScan methods. The ROR and PRR methods detected rather too many signals, even if the number reported was small. Thus, the restriction of three or more cases for the reported count to be a signal for the ROR and PRR methods, which is usually imposed in practice [3], might be sensible.

In terms of computation time, the GPS, LRT, and TreeScan methods are more intensive relative to the other methods. Other methods have a closed form for the confidence interval of each statistic, so only the cell count ($n_{ij}$) and marginal count ($n_{i.}$ or $n_{.j}$) of the matrix are required to calculate the confidence interval. On the other hand, the GPS method requires all cell counts in the matrix to estimate the parameters of prior distribution. For the LRT and TreeScan method, a Monte Carlo simulation is required to obtain *p*-values.

The methods considered in this paper are approaches that can be applied to an existing database. In some cases, one may want to continuously or sequentially monitor to detect a signal as early as possible. The sequential probability ratio test (SPRT) [21,22] can be used. The method has also been applied to a spontaneous adverse event reporting system [23,24]. However, the result of the SPRT method is highly dependent on the relative risk used to specify the alternative hypothesis [25]. Although we did not include the SPRT in this study for these reasons, it would be interesting to compare the method in appropriate situations in future research.

The drug safety databases such as KAERS are constructed by a spontaneous reporting system and very few AEs that occur were reported, so it has a large number of zero-count cells. In this situation, a zero-inflated Poisson model could be considered. Hu et al. [11] proposed ZIP-sB and ZIP-DP (Dirichlet process). Huang et al. [26] proposed a zero-inflated Poisson (ZIP) model based on the likelihood ratio test. According to these research findings, ZIP models detected fewer signals in data containing a large number of zero-counts. This means that they are more conservative by considering zero-counts. In a further study, we will evaluate the performance of ZIP models and apply them to real data to compare.

Huang et al. proposed extending the likelihood ratio test-based (LRT) methods [9] that can detect signals for including a single AE or several AEs within one AE group. The extended LRT method could be used for hierarchical structures of AEs for a fixed drug. The threshold for a signal for multiple-layer analysis should be higher than that for single-layer analysis. It will be very interesting to see the simulation results by comparing the Extended LRT vs. TreeScan with multiple layers (PT, SOC, or others). This is a future research topic.

Currently, some drug companies have different AE detection criteria. For example, AstraZeneca detects an AE when the EB05 is greater than 1.8, whereas GlaxoSmithKline detects AE when it is greater than 2 [12]. In our study, it was confirmed that the performance of each method could vary depending on the cutoff, which is the criteria for signal detection in simulation. Therefore, how to set the cutoff for signal detection is very important and worth noting.

## 6. Conclusions

In summary, the LRT, GPS, BCPNN, sB, and TreeScan methods are more conservative than the ROR, PRR, and IC methods. Only the TreeScan method controls the type I error rate at the desired level. No method is superior to the others in relation to all performance measures. It is recommended that those conducting drug‒AE surveillance use not just one method, but make a decision based on several methods.

## Figures and Tables

**Table 1:** Adverse events count for the *i*th adverse event and the *j*th drug.

AE	jth Drug	All Other Drugs	Total
***i*th** **adverse event**	$n_{ij}$	$n_{i.}-n_{ij}$	$n_{i.}$
**All other adverse events**	$n_{.j}-n_{ij}$	$n_{..}-n_{i.}-n_{.j}+n_{ij}$	$n_{..}-n_{i.}$
**Total**	$n_{.j}$	$n_{..}-n_{.j}$	$n_{..}$

**Table 2:** Comparison of type I error rates at various cutoff points when rr=1.

Total Sample Size		300,000	500,000	1,000,000
Method	Cutoff *	Type I Error		
ROR	1	1.000	1.000	0.999
	1.5	1.000	0.999	0.991
	2	0.999	0.979	0.914
	2.5	0.978	0.931	0.790
	3	0.939	0.861	0.679
PRR	1	1.000	1.000	0.999
	1.5	1.000	0.998	0.991
	2	0.999	0.978	0.910
	2.5	0.974	0.929	0.786
	3	0.934	0.860	0.676
IC	$\log_{2}\left(1\right)$	0.995	0.998	1.000
	$\log_{2}\left(1.5\right)$	0.992	0.994	0.993
	$\log_{2}\left(2\right)$	0.828	0.717	0.546
	$\log_{2}\left(2.5\right)$	0.607	0.499	0.335
	$\log_{2}\left(3\right)$	0.284	0.212	0.121
LRT	0.2	0.241	0.215	0.207
	0.1	0.124	0.107	0.117
	0.05	0.068	0.063	0.053
	0.025	0.044	0.039	0.029
	0.01	0.031	0.012	0.020
GPS	1	0.567	0.615	0.656
	1.5	0.009	0.010	0.005
	2	0.000	0.000	0.000
	2.5	0.000	0.000	0.000
	3	0.000	0.000	0.000
BCPNN	$\log_{2}\left(1\right)$	0.371	0.949	0.959
	$\log_{2}\left(1.5\right)$	0.024	0.113	0.078
	$\log_{2}\left(2\right)$	0.000	0.003	0.003
	$\log_{2}\left(2.5\right)$	0.000	0.000	0.001
	$\log_{2}\left(3\right)$	0.000	0.000	0.000
sB	1	0.741	0.842	0.917
	1.5	0.088	0.090	0.079
	2	0.009	0.005	0.004
	2.5	0.000	0.000	0.000
	3	0.000	0.000	0.000
TreeScan	0.2	0.194	0.240	0.219
	0.1	0.103	0.124	0.097
	0.05	0.052	0.050	0.047
	0.025	0.025	0.029	0.029
	0.01	0.008	0.010	0.009

ROR, Reporting Odds Ratio; PRR, Proportional Reporting Ratio; IC, Information Component; LRT, Likelihood ratio test; GPS, Gamma Poisson Shrinker; BCPNN, Bayesian Confidence Propagation Neural Network; sB simplified Bayes; TreeScan, Tree-based Scan Statistic; * Cutoff values for the lower bound of the 95% CI for ROR, PRR, IC, BCPNN, and sB, for EB05 for GPS, and for the *p*-value for LRT and TreeScan.

**Table 3:** Summary of performance for each method at various cutoff points when the total sample size = 300,000 and $rr\sim U\left(1.2,\;10\right)$.

True Signal Ratio		0.03			0.05			0.1		
Method	Cutoff *	Power	Sensitivity	PPV	Power	Sensitivity	PPV	Power	Sensitivity	PPV
ROR	1	0.996	0.753	0.251	0.997	0.728	0.394	0.999	0.680	0.649
	1.5	0.996	0.704	0.388	0.997	0.678	0.534	0.999	0.621	0.744
	2	0.996	0.655	0.503	0.997	0.624	0.628	0.999	0.561	0.800
	2.5	0.996	0.608	0.587	0.997	0.569	0.692	0.999	0.498	0.833
	3	0.996	0.559	0.646	0.997	0.519	0.737	0.999	0.438	0.854
PRR	1	0.996	0.753	0.251	0.997	0.728	0.394	0.999	0.680	0.649
	1.5	0.996	0.704	0.389	0.997	0.678	0.535	0.999	0.621	0.744
	2	0.996	0.654	0.504	0.997	0.623	0.629	0.999	0.560	0.800
	2.5	0.996	0.607	0.588	0.997	0.568	0.693	0.999	0.497	0.833
	3	0.996	0.557	0.647	0.997	0.516	0.738	0.999	0.436	0.855
IC	$\log_{2}\left(1\right)$	0.991	0.687	0.309	0.995	0.660	0.479	0.995	0.613	0.748
	$\log_{2}\left(1.5\right)$	0.986	0.612	0.614	0.991	0.579	0.757	0.992	0.526	0.904
	$\log_{2}\left(2\right)$	0.980	0.541	0.825	0.984	0.507	0.881	0.990	0.448	0.951
	$\log_{2}\left(2.5\right)$	0.976	0.500	0.877	0.982	0.467	0.917	0.989	0.405	0.963
	$\log_{2}\left(3\right)$	0.963	0.413	0.938	0.977	0.375	0.956	0.986	0.311	0.978
LRT	0.2	0.939	0.462	0.962	0.956	0.417	0.983	0.973	0.338	0.990
	0.1	0.929	0.432	0.981	0.945	0.388	0.990	0.969	0.312	0.995
	0.05	0.915	0.409	0.990	0.930	0.365	0.992	0.961	0.289	0.996
	0.025	0.901	0.387	0.994	0.922	0.341	0.995	0.947	0.267	1.000
	0.01	0.881	0.359	0.997	0.908	0.314	0.999	0.932	0.240	1.000
GPS	1	0.891	0.417	0.997	0.926	0.395	0.998	0.951	0.378	0.997
	1.5	0.888	0.415	0.998	0.925	0.393	0.998	0.951	0.378	0.997
	2	0.888	0.414	0.998	0.925	0.391	0.998	0.950	0.369	0.998
	2.5	0.888	0.406	0.998	0.924	0.373	0.998	0.945	0.312	1.000
	3	0.886	0.369	0.999	0.911	0.315	0.999	0.913	0.204	1.000
BCPNN	$\log_{2}\left(1\right)$	0.948	0.578	0.731	0.951	0.539	0.857	0.972	0.474	0.957
	$\log_{2}\left(1.5\right)$	0.914	0.443	0.984	0.924	0.398	0.992	0.950	0.323	0.998
	$\log_{2}\left(2\right)$	0.867	0.335	0.998	0.893	0.291	0.999	0.911	0.216	1.000
	$\log_{2}\left(2.5\right)$	0.808	0.246	1.000	0.831	0.204	1.000	0.837	0.139	1.000
	$\log_{2}\left(3\right)$	0.719	0.174	1.000	0.753	0.138	1.000	0.754	0.081	1.000
sB	1	0.934	0.488	0.866	0.942	0.448	0.939	0.939	0.387	0.988
	1.5	0.921	0.414	0.992	0.932	0.363	0.996	0.919	0.293	0.999
	2	0.900	0.347	1.000	0.908	0.292	1.000	0.894	0.217	1.000
	2.5	0.864	0.288	1.000	0.875	0.231	1.000	0.856	0.156	1.000
	3	0.807	0.241	1.000	0.826	0.180	1.000	0.797	0.110	1.000
TreeScan	0.2	0.942	0.477	0.964	0.954	0.444	0.981	0.975	0.369	0.992
	0.1	0.930	0.457	0.983	0.949	0.417	0.991	0.968	0.343	0.996
	0.05	0.917	0.437	0.990	0.942	0.393	0.997	0.955	0.322	0.998
	0.025	0.904	0.422	0.994	0.935	0.373	0.999	0.948	0.301	1.000
	0.01	0.887	0.400	0.997	0.914	0.352	0.999	0.924	0.280	1.000

ROR, Reporting Odds Ratio; PRR, Proportional Reporting Ratio; IC, Information Component; LRT, Likelihood ratio test; GPS, Gamma Poisson Shrinker; BCPNN, Bayesian Confidence Propagation Neural Network; sB simplified Bayes; TreeScan, Tree-based Scan Statistic; * Cutoff values for the lower bound of the 95% CI for ROR, PRR, IC, BCPNN, and sB, for EB05 for GPS, and for the *p*-value for LRT and TreeScan.

**Table 4:** Summary of performance for each method at various cutoff points when the total sample size = 300,000 and $rr\sim U\left(1.2,\;4\right)$.

True Signal Ratio		0.03			0.05			0.1		
Method	Cutoff *	Power	Sensitivity	PPV	Power	Sensitivity	PPV	Power	Sensitivity	PPV
ROR	1	0.987	0.518	0.164	0.997	0.510	0.262	0.995	0.464	0.437
	1.5	0.984	0.419	0.250	0.997	0.408	0.364	0.995	0.362	0.531
	2	0.971	0.323	0.316	0.996	0.313	0.437	0.995	0.268	0.581
	2.5	0.934	0.243	0.348	0.984	0.231	0.468	0.994	0.190	0.597
	3	0.864	0.177	0.353	0.943	0.163	0.467	0.984	0.133	0.591
PRR	1	0.987	0.518	0.164	0.997	0.510	0.262	0.995	0.464	0.437
	1.5	0.984	0.419	0.250	0.997	0.408	0.364	0.995	0.361	0.532
	2	0.971	0.322	0.317	0.996	0.312	0.437	0.995	0.267	0.582
	2.5	0.933	0.241	0.349	0.984	0.229	0.469	0.994	0.189	0.597
	3	0.861	0.175	0.354	0.940	0.162	0.467	0.983	0.132	0.591
IC	$\log_{2}\left(1\right)$	0.944	0.472	0.182	0.984	0.469	0.290	0.984	0.413	0.486
	$\log_{2}\left(1.5\right)$	0.901	0.334	0.378	0.969	0.331	0.527	0.976	0.284	0.702
	$\log_{2}\left(2\right)$	0.835	0.222	0.582	0.942	0.217	0.696	0.965	0.179	0.811
	$\log_{2}\left(2.5\right)$	0.782	0.175	0.662	0.905	0.165	0.759	0.946	0.129	0.850
	$\log_{2}\left(3\right)$	0.569	0.087	0.744	0.716	0.077	0.820	0.828	0.056	0.882
LRT	0.2	0.673	0.149	0.867	0.785	0.136	0.917	0.859	0.111	0.955
	0.1	0.602	0.122	0.911	0.725	0.111	0.948	0.815	0.089	0.981
	0.05	0.554	0.104	0.939	0.670	0.091	0.975	0.748	0.071	0.988
	0.025	0.509	0.088	0.966	0.609	0.077	0.984	0.679	0.057	0.992
	0.01	0.444	0.071	0.977	0.534	0.060	0.987	0.612	0.044	0.999
GPS	1	0.430	0.079	0.983	0.611	0.095	0.989	0.704	0.090	0.992
	1.5	0.051	0.008	1.000	0.250	0.026	0.996	0.561	0.047	0.999
	2	0.028	0.005	1.000	0.165	0.017	1.000	0.465	0.034	0.999
	2.5	0.015	0.002	1.000	0.065	0.005	1.000	0.057	0.002	1.000
	3	0.003	0.000	1.000	0.008	0.001	1.000	0.003	0.000	1.000
BCPNN	$\log_{2}\left(1\right)$	0.864	0.306	0.477	0.914	0.292	0.624	0.943	0.259	0.802
	$\log_{2}\left(1.5\right)$	0.640	0.135	0.915	0.745	0.121	0.937	0.824	0.098	0.976
	$\log_{2}\left(2\right)$	0.339	0.048	0.984	0.426	0.040	0.996	0.507	0.028	1.000
	$\log_{2}\left(2.5\right)$	0.113	0.013	1.000	0.140	0.010	1.000	0.166	0.006	1.000
	$\log_{2}\left(3\right)$	0.022	0.003	1.000	0.026	0.002	1.000	0.025	0.001	1.000
sB	1	0.669	0.171	0.860	0.743	0.161	0.927	0.778	0.133	0.969
	1.5	0.472	0.080	0.991	0.569	0.072	0.995	0.628	0.052	0.997
	2	0.247	0.031	0.996	0.294	0.024	1.000	0.345	0.015	1.000
	2.5	0.084	0.009	1.000	0.094	0.006	1.000	0.098	0.003	1.000
	3	0.016	0.002	1.000	0.021	0.001	1.000	0.018	0.001	1.000
TreeScan	0.2	0.671	0.148	0.866	0.772	0.137	0.919	0.843	0.110	0.945
	0.1	0.619	0.125	0.930	0.726	0.114	0.965	0.792	0.088	0.964
	0.05	0.573	0.107	0.961	0.671	0.096	0.982	0.729	0.073	0.983
	0.025	0.520	0.092	0.970	0.614	0.082	0.990	0.685	0.061	0.991
	0.01	0.452	0.074	0.985	0.550	0.065	0.993	0.609	0.048	0.995

ROR, Reporting Odds Ratio; PRR, Proportional Reporting Ratio; IC, Information Component; LRT, Likelihood ratio test; GPS, Gamma Poisson Shrinker; BCPNN, Bayesian Confidence Propagation Neural Network; sB simplified Bayes; TreeScan, Tree-based Scan Statistic; * Cutoff values for the lower bound of the 95% CI for ROR, PRR, IC, BCPNN, and sB, for EB05 for GPS, and for the *p*-value for LRT and TreeScan.

**Table 5:** Causality assessment criteria.

Criterion	Level
The context of administration and use of medicines is reasonable.	Certain, Probable, Possible
It is not described as another medication, chemical, or accompanying illness.	Certain, Probable
In case of administration interruption, there is a clinically reasonable response.	Certain, Probable
In case of readministration, there is a pharmacologically conclusive response.	Certain
It could be described as another medication, chemical, or accompanying illness.	Possible, Unlikely
It is a temporary condition, not related to the administration and use of medicines.	Unlikely
It requires more information to assess or it is under examination.	Unclassified
It is not assessable and cannot be supplemented.	Unassessable

**Table 6:** Subset of WHO-ART’s hierarchical structure of adverse events.

Code	Level	Adverse Event
100	SOC	Skin and appendages disorders
100.0001.001	PT	ACNE
100.0001.003	IT	ACNEIFORM DERMATITIS
100.0001.004	IT	RASH ACNEIFORM
100.0001.005	IT	ACNE CYSTIC
100.0001.006	IT	ACNE PUSTULAR
100.0001.007	IT	ACNE AGGRAVATED
100.0001.008	IT	ACNE CONGLOBATA
100.0002.001	PT	ALOPECIA
100.0002.003	IT	HAIR THINNING
100.0002.004	IT	ALOPECIA AREATA
100.0002.005	IT	ATRICHIA
100.0002.006	IT	BALDNESS
100.0002.007	IT	HAIR LOSS
100.0002.008	IT	ATRICHOSIS
100.0002.009	IT	LOSS OF EYELASHES
100.0002.010	IT	ALOPECIA TOTALIS
100.0002.011	IT	ALOPECIA SCARRING
100.0002.012	IT	ALOPECIA UNIVERSALIS
100.0002.013	IT	DEFLUVIUM
100.0002.014	IT	LOSS OF EYEBROWS
100.0002.015	IT	AGGRAVATED HAIR LOSS

**Table 7:** Signal detection criterion for each method.

Method	Detection Criterion
ROR, PRR	95% CI lower bound > 2
IC, BCPNN	95% CI lower bound > log2(2)
GPS	EB05 > 2
BCPNN	95% CI lower bound > log2(2)
sB	95% CI lower bound > 2
LRT, TreeScan	*p*-value < 0.05

ROR, Reporting Odds Ratio; PRR, Proportional Reporting Ratio; IC, Information Component; LRT, Likelihood ratio test; GPS, Gamma Poisson Shrinker; BCPNN, Bayesian Confidence Propagation Neural Network; sB simplified Bayes; TreeScan, Tree-based Scan Statistic.

**Table 8:** Overall detection: the number of signals detected by each method in the 2012–2016 Korea adverse event reporting system (KAERS) database contained 1615 kinds of adverse events and 1716 kinds of drugs.

Method (# of Pairs)	ROR & PRR	IC	LRT	GPS	BCPNN	sB	TreeScan
PT levels (2,546,544)	43,960	25,714	8324	6147	5290	4397	9175
SOC levels (54,912)	4142	2147	2238	1342	1256	1163	1380
Total (2,601,456)	48,102	27,861	10,562	7489	6546	5560	10,555

ROR, Reporting Odds Ratio; PRR, Proportional Reporting Ratio; IC, Information Component; LRT, Likelihood ratio test; GPS, Gamma Poisson Shrinker; BCPNN, Bayesian Confidence Propagation Neural Network; sB simplified Bayes; TreeScan, Tree-based Scan Statistic.

**Table 9:** Detected signals by each method for voglibose and acarbose.

		Adverse Event	Obs	Exp	ROR	PRR	IC	LRT	GPS	BCPNN	sB	TreeScan
Voglibose	500_165	Anorexia	8	2.62	2.42 *	2.42 *	0.59	0.940	1.12	0.35	1.37	0.504
	600	Gastrointestinal system disorders	115	73.73	1.83	1.83	0.32	0.001 *	1.28	0.31	1.46	0.001 *
	600_204	Constipation	11	3.96	2.26 *	2.26 *	0.60	0.782	1.24	0.43	1.47	0.336
	600_205	Diarrhea	12	6.43	1.33	1.33	0.06	1.000	0.92	0.00	1.23	0.980
	600_268	Abdominal pain	10	3.63	2.20 *	2.20 *	0.55	0.910	1.17	0.37	1.43	0.447
	600_279	Dyspepsia	35	7.22	5.16 *	5.15 *	1.77 *	0.001 *	3.11 *	1.62 *	3.64 *	0.001 *
	600_285	Flatulence	15	0.39	40.28 *	40.00 *	4.49 *	0.001 *	20.89 *	2.79 *	10.76 *	0.001 *
	800	Metabolic and nutritional disorders	37	5.07	8.11 *	8.10 *	2.37 *	0.001 *	4.67 *	2.15 *	6.11 *	0.001 *
	800_389	Hypoglycemia	24	0.55	48.25 *	47.86 *	4.84 *	0.001 *	27.47 *	3.41 *	17.12 *	0.001 *
	800_392	Hyponatremia	2	0.18	9.67 *	9.65 *	1.44 *	0.996	0.49	−0.30	0.03	0.772
	800_407	Weight decrease	2	0.21	8.19 *	8.18 *	1.24 *	0.998	0.47	−0.34	0.04	0.860
	1100	Respiratory system disorders	16	9.46	1.23	1.23	0.03	1.000	0.94	−0.02	1.21	0.981
	1100_515	Epistaxis	2	0.21	8.18 *	8.17 *	1.24 *	0.998	0.47	−0.34	0.04	0.861
	1100_523	Pharyngitis	4	0.85	3.79 *	3.79 *	0.81	0.992	0.85	0.15	0.80	0.745
	1810_401	Edema peripheral	3	0.71	3.15 *	3.15 *	0.44	1.000	0.60	−0.20	0.43	0.973
Acarbose	500_172	Depression	2	0.08	25.07 *	25.02 *	2.68 *	0.638	0.66	−0.18	0.04	0.209
	600_205	Diarrhea	7	3.01	1.65	1.64	0.11	1.000	0.84	−0.05	0.90	0.924
	600_285	Flatulence	12	0.18	72.43 *	72.02 *	5.16 *	0.001 *	31.93 *	2.62 *	10.06 *	0.001 *
	600_336	Tooth disorder	2	0.01	180.65 *	177.84 *	5.43 *	0.010 *	1.88	−0.10	0.02	0.008 *
	800	Metabolic and nutritional disorders	6	2.38	1.79	1.79	0.15	1.000	0.81	−0.06	0.89	0.920
	800_383	Hyperkalemia	2	0.11	17.08 *	17.06 *	2.16 *	0.846	0.58	−0.22	0.03	0.390
	800_389	Hypoglycemia	3	0.26	10.77 *	10.76 *	1.88 *	0.632	0.91	0.23	0.64	0.202
	1210	Red blood cell disorders	4	0.62	5.70 *	5.70 *	1.26 *	0.798	1.00	0.33	0.98	0.326
	1210_544	Anemia	4	0.51	7.13 *	7.13 *	1.54 *	0.556	1.10	0.43	1.05	0.176
	1300	Urinary system disorders	6	2.16	2.06 *	2.06 *	0.29	0.998	0.87	0.04	0.91	0.819
	1300_619	Renal function abnormal	2	0.11	16.52 *	16.50 *	2.12 *	0.860	0.57	−0.23	0.03	0.396
	1810_711	Abdomen enlarged	2	0.08	25.76 *	25.71 *	2.72 *	0.632	0.66	−0.18	0.02	0.197

Obs, Observed count; Exp, Expected count; ROR, Reporting Odds Ratio; PRR, Proportional Reporting Ratio; IC, Information Component; LRT, Likelihood ratio test; GPS, Gamma Poisson Shrinker; BCPNN, Bayesian Confidence Propagation Neural Network; sB simplified Bayes; TreeScan, Tree-based Scan Statistic; * signal.

## Appendix A

**Table A1:** Summary of performance for each method at various cutoff points when the total sample size = 500,000 and $rr\sim U\left(1.2,\;10\right)$.

True Signal Ratio		0.03			0.05			0.1		
Method	Cutoff *	Power	Sensitivity	PPV	Power	Sensitivity	PPV	Power	Sensitivity	PPV
ROR	1	0.996	0.799	0.303	0.995	0.764	0.462	0.998	0.728	0.729
	1.5	0.996	0.747	0.493	0.995	0.709	0.631	0.998	0.663	0.821
	2	0.995	0.696	0.621	0.995	0.651	0.726	0.998	0.596	0.865
	2.5	0.994	0.641	0.694	0.995	0.595	0.780	0.998	0.532	0.888
	3	0.994	0.590	0.740	0.995	0.539	0.813	0.998	0.465	0.904
PRR	1	0.996	0.799	0.303	0.995	0.764	0.462	0.998	0.728	0.729
	1.5	0.996	0.747	0.495	0.995	0.708	0.631	0.998	0.663	0.821
	2	0.995	0.695	0.622	0.995	0.650	0.727	0.998	0.596	0.865
	2.5	0.994	0.639	0.695	0.995	0.593	0.781	0.998	0.530	0.889
	3	0.994	0.588	0.741	0.995	0.537	0.813	0.998	0.462	0.904
IC	$\log_{2}\left(1\right)$	0.993	0.758	0.285	0.994	0.735	0.468	0.992	0.682	0.760
	$\log_{2}\left(1.5\right)$	0.992	0.689	0.663	0.992	0.658	0.810	0.991	0.593	0.927
	$\log_{2}\left(2\right)$	0.990	0.619	0.866	0.992	0.586	0.922	0.987	0.512	0.967
	$\log_{2}\left(2.5\right)$	0.983	0.583	0.910	0.988	0.540	0.944	0.987	0.466	0.978
	$\log_{2}\left(3\right)$	0.975	0.494	0.952	0.987	0.446	0.973	0.984	0.366	0.985
LRT	0.2	0.958	0.558	0.962	0.974	0.527	0.989	0.974	0.440	0.994
	0.1	0.955	0.535	0.979	0.967	0.502	0.994	0.966	0.414	0.997
	0.05	0.946	0.515	0.988	0.961	0.477	0.997	0.959	0.392	1.000
	0.025	0.938	0.495	0.995	0.955	0.456	0.999	0.959	0.370	1.000
	0.01	0.935	0.471	0.999	0.944	0.433	0.999	0.949	0.344	1.000
GPS	1	0.926	0.511	0.998	0.934	0.493	0.999	0.968	0.478	0.999
	1.5	0.925	0.509	0.998	0.934	0.491	0.999	0.968	0.476	0.999
	2	0.925	0.506	0.999	0.934	0.482	0.999	0.967	0.447	0.999
	2.5	0.922	0.487	0.999	0.932	0.439	0.999	0.965	0.355	0.999
	3	0.918	0.430	0.999	0.926	0.353	1.000	0.947	0.242	1.000
sB	1	0.940	0.593	0.845	0.962	0.552	0.937	0.961	0.491	0.987
	1.5	0.929	0.505	0.990	0.948	0.462	0.997	0.954	0.388	0.999
	2	0.910	0.426	0.998	0.938	0.373	1.000	0.948	0.301	1.000
	2.5	0.880	0.362	1.000	0.920	0.298	1.000	0.934	0.226	1.000
	3	0.841	0.310	1.000	0.889	0.234	1.000	0.902	0.162	1.000
BCPNN	$\log_{2}\left(1\right)$	0.964	0.670	0.737	0.972	0.646	0.870	0.972	0.568	0.964
	$\log_{2}\left(1.5\right)$	0.947	0.542	0.982	0.960	0.509	0.996	0.958	0.422	0.999
	$\log_{2}\left(2\right)$	0.930	0.435	1.000	0.936	0.396	1.000	0.937	0.305	1.000
	$\log_{2}\left(2.5\right)$	0.893	0.342	1.000	0.907	0.299	1.000	0.914	0.215	1.000
	$\log_{2}\left(3\right)$	0.837	0.268	1.000	0.875	0.223	1.000	0.865	0.142	1.000
TreeScan	0.2	0.956	0.569	0.970	0.960	0.525	0.981	0.981	0.464	0.996
	0.1	0.951	0.550	0.985	0.957	0.504	0.992	0.976	0.440	0.997
	0.05	0.942	0.534	0.990	0.951	0.485	0.995	0.968	0.419	0.999
	0.025	0.932	0.519	0.996	0.940	0.471	0.998	0.961	0.400	1.000
	0.01	0.921	0.495	0.999	0.929	0.450	1.000	0.957	0.376	1.000

ROR, Reporting Odds Ratio; PRR, Proportional Reporting Ratio; IC, Information Component; LRT, Likelihood ratio test; GPS, Gamma Poisson Shrinker; BCPNN, Bayesian Confidence Propagation Neural Network; sB simplified Bayes; TreeScan, Tree-based Scan Statistic; * Cutoff values for the lower bound of the 95% CI for ROR, PRR, IC, BCPNN, and sB, for EB05 for GPS, and for the *p*-value for LRT and TreeScan.

**Table A2:** Summary of performance for each method at various cutoff points when the total sample size = 1,000,000 and $rr\sim U\left(1.2,\;10\right)$.

True Signal Ratio		0.03			0.05			0.1		
Method	Cutoff *	Power	Sensitivity	PPV	Power	Sensitivity	PPV	Power	Sensitivity	PPV
ROR	1	0.997	0.853	0.391	0.996	0.829	0.569	1.000	0.779	0.810
	1.5	0.997	0.800	0.649	0.996	0.772	0.755	1.000	0.711	0.886
	2	0.997	0.745	0.763	0.996	0.710	0.834	1.000	0.645	0.916
	2.5	0.997	0.691	0.818	0.996	0.645	0.871	1.000	0.575	0.932
	3	0.997	0.640	0.852	0.996	0.587	0.892	1.000	0.502	0.941
PRR	1	0.997	0.853	0.391	0.996	0.829	0.569	1.000	0.779	0.810
	1.5	0.997	0.800	0.650	0.996	0.772	0.755	1.000	0.711	0.886
	2	0.997	0.745	0.763	0.996	0.709	0.835	1.000	0.644	0.916
	2.5	0.997	0.691	0.819	0.996	0.645	0.871	1.000	0.574	0.932
	3	0.997	0.638	0.852	0.996	0.585	0.893	1.000	0.501	0.941
IC	$\log_{2}\left(1\right)$	0.994	0.837	0.263	0.992	0.817	0.451	1.000	0.777	0.794
	$\log_{2}\left(1.5\right)$	0.992	0.766	0.748	0.990	0.739	0.859	1.000	0.686	0.960
	$\log_{2}\left(2\right)$	0.989	0.694	0.912	0.988	0.663	0.954	1.000	0.600	0.983
	$\log_{2}\left(2.5\right)$	0.986	0.651	0.943	0.988	0.622	0.967	0.998	0.550	0.987
	$\log_{2}\left(3\right)$	0.983	0.556	0.971	0.987	0.525	0.983	0.994	0.438	0.991
LRT	0.2	0.969	0.664	0.982	0.982	0.642	0.992	0.988	0.571	0.997
	0.1	0.966	0.645	0.992	0.978	0.623	0.996	0.986	0.550	0.999
	0.05	0.959	0.625	0.996	0.971	0.605	1.000	0.982	0.532	0.999
	0.025	0.953	0.607	0.998	0.971	0.589	1.000	0.977	0.513	0.999
	0.01	0.949	0.584	1.000	0.967	0.567	1.000	0.972	0.492	1.000
GPS	1	0.954	0.641	0.998	0.967	0.621	0.999	0.980	0.590	0.999
	1.5	0.954	0.639	0.998	0.967	0.619	0.999	0.979	0.583	0.999
	2	0.953	0.625	0.998	0.967	0.587	0.999	0.978	0.524	1.000
	2.5	0.953	0.577	0.998	0.965	0.522	1.000	0.974	0.422	1.000
	3	0.952	0.501	0.998	0.964	0.428	1.000	0.963	0.314	1.000
sB	1	0.976	0.756	0.753	0.983	0.743	0.892	0.990	0.683	0.977
	1.5	0.958	0.640	0.991	0.968	0.617	0.998	0.976	0.545	1.000
	2	0.941	0.537	1.000	0.962	0.515	1.000	0.970	0.426	1.000
	2.5	0.921	0.450	1.000	0.949	0.420	1.000	0.958	0.324	1.000
	3	0.889	0.372	1.000	0.938	0.334	1.000	0.939	0.237	1.000
BCPNN	$\log_{2}\left(1\right)$	0.970	0.716	0.825	0.971	0.681	0.937	0.968	0.609	0.989
	$\log_{2}\left(1.5\right)$	0.961	0.635	0.994	0.966	0.583	0.997	0.961	0.508	1.000
	$\log_{2}\left(2\right)$	0.953	0.545	1.000	0.962	0.491	0.999	0.955	0.411	1.000
	$\log_{2}\left(2.5\right)$	0.941	0.472	1.000	0.955	0.409	1.000	0.943	0.326	1.000
	$\log_{2}\left(3\right)$	0.926	0.406	1.000	0.948	0.335	1.000	0.932	0.247	1.000
TreeScan	0.2	0.972	0.688	0.980	0.979	0.651	0.992	0.985	0.574	0.999
	0.1	0.966	0.673	0.988	0.977	0.632	0.996	0.983	0.553	0.999
	0.05	0.964	0.658	0.992	0.974	0.615	0.997	0.983	0.533	1.000
	0.025	0.959	0.646	0.996	0.966	0.603	1.000	0.977	0.518	1.000
	0.01	0.955	0.627	0.997	0.963	0.581	1.000	0.968	0.500	1.000

ROR, Reporting Odds Ratio; PRR, Proportional Reporting Ratio; IC, Information Component; LRT, Likelihood ratio test; GPS, Gamma Poisson Shrinker; BCPNN, Bayesian Confidence Propagation Neural Network; sB simplified Bayes; TreeScan, Tree-based Scan Statistic; * Cutoff values for the lower bound of the 95% CI for ROR, PRR, IC, BCPNN, and sB, for EB05 for GPS, and for the *p*-value for LRT and TreeScan.

**Table A3:** Summary of performance for each method at various cutoff points when the total sample size = 500,000 and $rr\sim U\left(1.2,\;4\right)$.

True Signal Ratio		0.03			0.05			0.1		
Method	Cutoff *	Power	Sensitivity	PPV	Power	Sensitivity	PPV	Power	Sensitivity	PPV
ROR	1	0.991	0.592	0.204	0.997	0.571	0.313	0.996	0.522	0.510
	1.5	0.989	0.471	0.341	0.997	0.452	0.465	0.996	0.403	0.637
	2	0.979	0.360	0.434	0.996	0.340	0.547	0.996	0.294	0.686
	2.5	0.954	0.260	0.465	0.989	0.238	0.576	0.996	0.198	0.693
	3	0.859	0.174	0.453	0.948	0.158	0.564	0.990	0.128	0.673
PRR	1	0.991	0.592	0.204	0.997	0.571	0.313	0.996	0.522	0.510
	1.5	0.989	0.470	0.342	0.997	0.451	0.465	0.996	0.403	0.638
	2	0.978	0.359	0.434	0.996	0.339	0.548	0.996	0.292	0.686
	2.5	0.954	0.258	0.466	0.989	0.237	0.576	0.996	0.197	0.694
	3	0.852	0.172	0.450	0.944	0.156	0.563	0.989	0.126	0.673
IC	$\log_{2}\left(1\right)$	0.968	0.578	0.177	0.979	0.556	0.281	0.987	0.511	0.493
	$\log_{2}\left(1.5\right)$	0.948	0.430	0.457	0.966	0.402	0.592	0.981	0.358	0.771
	$\log_{2}\left(2\right)$	0.914	0.296	0.698	0.944	0.274	0.777	0.965	0.231	0.866
	$\log_{2}\left(2.5\right)$	0.880	0.229	0.777	0.922	0.210	0.829	0.956	0.171	0.897
	$\log_{2}\left(3\right)$	0.647	0.106	0.850	0.769	0.093	0.881	0.857	0.068	0.909
LRT	0.2	0.810	0.230	0.910	0.860	0.210	0.940	0.918	0.179	0.968
	0.1	0.763	0.202	0.951	0.827	0.183	0.966	0.889	0.154	0.982
	0.05	0.721	0.180	0.973	0.794	0.159	0.982	0.860	0.134	0.991
	0.025	0.684	0.161	0.987	0.769	0.140	0.991	0.833	0.117	0.996
	0.01	0.639	0.139	0.995	0.722	0.119	0.992	0.784	0.097	0.999
GPS	1	0.650	0.163	0.979	0.745	0.179	0.984	0.792	0.185	0.990
	1.5	0.164	0.025	0.994	0.360	0.047	1.000	0.529	0.047	1.000
	2	0.062	0.009	1.000	0.150	0.018	1.000	0.170	0.011	1.000
	2.5	0.019	0.002	1.000	0.056	0.004	1.000	0.016	0.001	1.000
	3	0.006	0.001	1.000	0.005	0.000	1.000	0.001	0.000	1.000
sB	1	0.917	0.421	0.511	0.942	0.386	0.652	0.969	0.351	0.833
	1.5	0.790	0.210	0.947	0.841	0.186	0.968	0.894	0.155	0.979
	2	0.533	0.090	0.999	0.607	0.071	0.997	0.709	0.055	0.999
	2.5	0.210	0.025	1.000	0.276	0.021	1.000	0.300	0.012	1.000
	3	0.051	0.005	1.000	0.056	0.003	1.000	0.048	0.002	1.000
BCPNN	$\log_{2}\left(1\right)$	0.807	0.279	0.860	0.841	0.260	0.924	0.851	0.222	0.980
	$\log_{2}\left(1.5\right)$	0.655	0.140	0.991	0.716	0.127	0.995	0.747	0.100	0.997
	$\log_{2}\left(2\right)$	0.384	0.057	0.999	0.502	0.051	0.999	0.548	0.033	1.000
	$\log_{2}\left(2.5\right)$	0.150	0.017	1.000	0.208	0.015	1.000	0.201	0.007	1.000
	$\log_{2}\left(3\right)$	0.044	0.004	1.000	0.049	0.003	1.000	0.029	0.001	1.000
TreeScan	0.2	0.815	0.230	0.918	0.855	0.214	0.945	0.891	0.181	0.965
	0.1	0.776	0.202	0.948	0.833	0.187	0.966	0.852	0.156	0.982
	0.05	0.736	0.177	0.971	0.792	0.162	0.978	0.820	0.135	0.989
	0.025	0.691	0.157	0.983	0.749	0.143	0.988	0.784	0.118	0.990
	0.01	0.632	0.133	0.992	0.693	0.120	0.991	0.748	0.098	0.994

ROR, Reporting Odds Ratio; PRR, Proportional Reporting Ratio; IC, Information Component; LRT, Likelihood ratio test; GPS, Gamma Poisson Shrinker; BCPNN, Bayesian Confidence Propagation Neural Network; sB simplified Bayes; TreeScan, Tree-based Scan Statistic; * Cutoff values for the lower bound of the 95% CI for ROR, PRR, IC, BCPNN, and sB, for EB05 for GPS, and for the *p*-value for LRT and TreeScan.

**Table A4:** Summary of performance for each method at various cutoff points when the total sample size = 1,000,000 and $rr\sim U\left(1.2,\;4\right)$.

True Signal Ratio		0.03			0.05			0.1		
Method	Cutoff *	Power	Sensitivity	PPV	Power	Sensitivity	PPV	Power	Sensitivity	PPV
ROR	1	0.993	0.677	0.266	0.996	0.662	0.401	0.999	0.629	0.633
	1.5	0.993	0.541	0.512	0.996	0.520	0.621	0.999	0.477	0.774
	2	0.985	0.406	0.607	0.996	0.382	0.692	0.999	0.337	0.818
	2.5	0.960	0.279	0.633	0.991	0.257	0.715	0.999	0.212	0.821
	3	0.860	0.175	0.611	0.950	0.153	0.694	0.979	0.120	0.795
PRR	1	0.993	0.677	0.266	0.996	0.662	0.401	0.999	0.629	0.633
	1.5	0.993	0.541	0.513	0.996	0.520	0.621	0.999	0.476	0.774
	2	0.985	0.403	0.608	0.996	0.380	0.692	0.999	0.336	0.818
	2.5	0.959	0.277	0.633	0.991	0.255	0.715	0.999	0.210	0.822
	3	0.855	0.172	0.610	0.945	0.149	0.694	0.978	0.117	0.793
IC	$\log_{2}\left(1\right)$	0.986	0.705	0.164	0.989	0.687	0.272	0.996	0.660	0.508
	$\log_{2}\left(1.5\right)$	0.975	0.537	0.585	0.981	0.517	0.704	0.994	0.479	0.856
	$\log_{2}\left(2\right)$	0.953	0.383	0.818	0.970	0.359	0.870	0.992	0.318	0.934
	$\log_{2}\left(2.5\right)$	0.932	0.300	0.869	0.957	0.277	0.906	0.986	0.234	0.947
	$\log_{2}\left(3\right)$	0.760	0.140	0.922	0.855	0.116	0.942	0.924	0.086	0.960
LRT	0.2	0.899	0.382	0.941	0.925	0.347	0.964	0.952	0.312	0.988
	0.1	0.885	0.357	0.964	0.907	0.316	0.985	0.941	0.284	0.995
	0.05	0.871	0.333	0.979	0.897	0.291	0.994	0.935	0.259	0.996
	0.025	0.855	0.310	0.992	0.885	0.270	0.998	0.918	0.236	0.999
	0.01	0.837	0.285	0.997	0.859	0.244	0.999	0.895	0.211	1.000
GPS	1	0.838	0.298	0.995	0.866	0.292	0.995	0.902	0.330	0.991
	1.5	0.813	0.262	0.999	0.841	0.253	0.999	0.811	0.177	0.999
	2	0.768	0.191	1.000	0.771	0.156	1.000	0.583	0.064	1.000
	2.5	0.362	0.049	1.000	0.372	0.032	1.000	0.136	0.006	1.000
	3	0.033	0.003	1.000	0.038	0.002	1.000	0.004	0.000	1.000
sB	1	0.961	0.562	0.561	0.963	0.525	0.707	0.967	0.491	0.872
	1.5	0.892	0.338	0.967	0.917	0.300	0.983	0.944	0.261	0.996
	2	0.764	0.179	1.000	0.797	0.138	1.000	0.855	0.106	0.999
	2.5	0.449	0.063	1.000	0.509	0.046	0.998	0.544	0.027	1.000
	3	0.124	0.013	1.000	0.132	0.009	1.000	0.121	0.004	1.000
BCPNN	$\log_{2}\left(1\right)$	0.915	0.441	0.860	0.929	0.417	0.921	0.927	0.383	0.979
	$\log_{2}\left(1.5\right)$	0.843	0.265	0.996	0.863	0.239	0.998	0.885	0.205	1.000
	$\log_{2}\left(2\right)$	0.655	0.131	1.000	0.719	0.112	1.000	0.788	0.083	1.000
	$\log_{2}\left(2.5\right)$	0.355	0.048	1.000	0.426	0.037	1.000	0.460	0.021	1.000
	$\log_{2}\left(3\right)$	0.094	0.010	1.000	0.114	0.007	1.000	0.086	0.003	1.000
TreeScan	0.2	0.913	0.378	0.956	0.932	0.346	0.967	0.955	0.313	0.986
	0.1	0.899	0.348	0.977	0.913	0.319	0.988	0.935	0.285	0.992
	0.05	0.879	0.323	0.988	0.896	0.297	0.991	0.910	0.261	0.995
	0.025	0.854	0.300	0.993	0.877	0.273	0.996	0.905	0.241	0.997
	0.01	0.834	0.273	0.997	0.846	0.245	0.999	0.885	0.215	0.997

ROR, Reporting Odds Ratio; PRR, Proportional Reporting Ratio; IC, Information Component; LRT, Likelihood ratio test; GPS, Gamma Poisson Shrinker; BCPNN, Bayesian Confidence Propagation Neural Network; sB simplified Bayes; TreeScan, Tree-based Scan Statistic; * Cutoff values for the lower bound of the 95% CI for ROR, PRR, IC, BCPNN, and sB, for EB05 for GPS, and for the *p*-value for LRT and TreeScan.

## References

[1] Korea Institution of Drug Safety & Risk Management (2017). Guideline for KIDS-Korea Adverse Event Reporting System Database.

[2] Rothman K.J., Lanes S., Sacks S.T. (2004). The reporting odds ratio and its advantages over the proportional reporting ratio. Pharmacoepidemiol. Drug Saf..

[3] Evans S.J., Waller P.C., Davis S. (2001). Use of proportional reporting ratios (PRRs) for signal generation from spontaneous adverse drug reaction reports. Pharmacoepidemiol. Drug Saf..

[4] Huang L., Zalkikar J., Tiwari R.C. (2011). A likelihood ratio test based method for signal detection with application to FDA’s drug safety data. J. Am. Stat. Assoc..

[5] Dumouchel W. (1999). Bayesian Data mining in large frequency tables, with an application to the FDA apontaneous reporting system. Am. Stat..

[6] Bate A., Lindquist M., Edwards I.R., Olsson S., Orre R., Lansner A., De Freitas R.M. (1998). A bayesian neural network method for adverse drug reaction signal generation. Eur. J. Clin. Pharmacol..

[7] Noren G.N., Bate A., Orre R., Edwards I.R. (2006). Extending the methods used to screen the WHO drug safety database towards analysis of complex associations and improved accuracy for rare events. Stat. Med..

[8] Norén G.N., Edwards I.R. (2010). Opportunities and challenges of adverse drug reaction surveillance in electronic patient records. Pharmacovigil. Rev..

[9] Huang L., Zalkikar J., Tiwari R.C. (2013). Likelihood ratio test-based method for signal detection in drug classes using FDA’s AERS database. J. Biopharm. Stat..

[10] Huang L., Guo T., Zalkikar J.N., Tiwari R.C. (2014). A review of statistical methods for safety surveillance. Ther. Innov. Regul. Sci..

[11] Hu N., Huang L., Tiwari R.C. (2015). Signal detection in FDA AERS database using Dirichlet process. Stat. Med..

[12] Candore G., Juhlin K., Manlik K., Thakrar B., Quarcoo N., Seabroke S., Wisniewski A., Slattery J. (2015). Comparison of statistical signal detection methods within and across spontaneous reporting databases. Drug Saf..

[13] Kulldorff M., Fang Z., Walsh S.J. (2003). A tree-based scan statistic for database disease surveillance. Biometrics.

[14] Kulldorff M., Dashevsky I., Avery T.R., Chan A.K., Davis R.L., Graham D., Platt R., Andrade S.E., Boudreau D., Gunter M. (2013). Drug safety data mining with a tree-based scan statistic. Pharmacoepidemiol. Drug Saf..

[15] Brown J.S., Petronis K.R., Bate A., Zhang F., Dashevsky I., Kulldorff M., Avery T.R., Davis R.L., Chan K.A., Andrade S.E. (2013). Drug adverse event detection in health plan data using the gamma poisson shrinker and comparison to the tree-based scan statistic. Pharmaceutics.

[16] (2015). The Uppsala Monitoring Centre: The WHO Adverse Reaction Terminology—WHO-ART, Terminology for Coding Clinical Information in Relation to Drug Therapy. https://www.who-umc.org/vigibase/services/learn-more-about-who-art/.

[17] Lee M.Y., Choi D.S., Lee M.K., Lee H.W., Park T.S., Kim D.M., Chung C.H., Kim D.K., Kim I.J., Jang H.C. (2014). Comparison of acarbose and voglibose in diabetes patients who are inadequately controlled with basal insulin treatment: Randomized, parallel, open-label, active-controlled study. J. Korean Med. Sci..

[18] Vichayanrat A., Ploybutr S., Tunlakit M., Watanakejorn P. (2002). Efficacy and safety of voglibose in comparison with acarbose in type 2 diabetic patients. Diabetes Res. Clin. Pract..

[19] Martin A.E., Montgomery P.A. (1996). Acarbose: An alpha-glucosidase inhibitor. Am. J. Health-Syst. Pharm. AJHP Off. J. Am. Soc. Health-Syst. Pharm..

[20] Dabhi A.S., Bhatt N.R., Shah M.J. (2013). Voglibose: An alpha glucosidase inhibitor. J. C. Diagn. Res. JCDR.

[21] Wald A. (1945). Sequential tests of statistical hypotheses. Ann. Math. Stat..

[22] Wald A. (1947). Sequential Analysis. Wald Sequential Analysis 1947.

[23] Chan C.L., Rudrappa S., San Ang P., Li S.C., Evans S.J. (2017). Detecting signals of disproportionate reporting from singapore’s spontaneous adverse event reporting system: An application of the sequential probability ratio test. Drug Saf..

[24] Chan C.L., Soh S., Tan S.H., Ang P.S., Rudrappa S., Li S.C., Evans S.J. (2020). Quantitative data mining in signal detection: The Singapore experience. Exp. Opin. Drug Saf..

[25] Kulldorff M., Davis R.L., Kolczak M., Lewis E., Lieu T., Platt R. (2011). A maximized sequential probability ratio test for drug and vaccine safety surveillance. Seq. Anal..

[26] Huang L., Zheng D., Zalkikar J., Tiwari R. (2017). Zero-inflated poisson model based likelihood ratio test for drug safety signal detection. Stat. Methods Med. Res..

